# *In vivo* optical-resolution optoacoustic flow cytometry in the oral mucosa using a transparent silicon-photonics acoustic detector (SPADE)^☆^

**DOI:** 10.1016/j.pacs.2026.100866

**Published:** 2026-07-30

**Authors:** Tamar Harary, Gil Gelbert, Ron Moisseev, Amir Rosenthal

**Affiliations:** The Andrew and Erna Viterbi Faculty of Electrical & Computer Engineering, Technion - Israel Institute of Technology, Haifa 3200003, Israel

**Keywords:** Optoacoustic, Optoacoustic flow cytometry, Optical-resolution optoacoustic microscopy, Optical-resolution photoacoustic microscopy, Silicon photonics acoustic detector, Transparent ultrasound detector, Microfluidics, Red blood cell counting, Capillary monitoring

## Abstract

Complete blood counts are central to clinical practice but require invasive sampling and centralized laboratory analysis. Optical-resolution photoacoustic microscopy provides intrinsic specificity to red blood cells (RBCs) through hemoglobin absorption. However, conventional implementations typically depend on bulky acoustic coupling schemes that hinder clinical translation. Here, we present a compact and integrable, label-free optoacoustic platform that combines optical-resolution excitation in an epi-illumination geometry with a transparent silicon-photonics acoustic detector (SPADE) featuring a wide acoustic acceptance angle that enables detection without strict optical–acoustic co-alignment. System performance was first characterized using optoacoustic point sources and subsequently evaluated in controlled RBC flow experiments within a tapered glass-capillary microfluidic phantom, as well as *in vivo* measurements of superficial inner-lip capillaries in human volunteers. The transparent SPADE preserved tight optical focusing (≤ 4 µm) and provided an acoustic bandwidth exceeding 100 MHz with a noise-equivalent pressure of approximately 2.3 mPa/Hz¹ ᐟ². Single-RBC transients were robustly detected in the phantom across varying flow conditions, while *in vivo* measurements at multiple capillary sites yielded reproducible transient signals consistent with individual RBC passages. These results establish a practical framework for stable, fixed-position optoacoustic detection of individual RBCs in superficial human capillaries, demonstrating the potential of transparent SPADE-based systems for non-invasive blood analysis.

## 1 Introduction

Complete blood counts are among the most frequently performed diagnostic tests in medicine, providing essential quantitative information on red blood cells (RBCs), white blood cells, and platelets for assessing anemia, infection, inflammation, hematologic malignancies, and overall patient health. Despite their ubiquity, conventional blood counts require invasive venipuncture and specialized laboratory processing, creating delays that limit real-time clinical decision-making. These constraints are particularly acute in emergency care, home-based monitoring, pediatrics, and low-resource environments, where rapid or repeated sampling is impractical. Accordingly, there is interest in new technologies capable of performing blood-cell analysis noninvasively and at the point of care, enabling immediate physiological feedback, reducing patient discomfort, and offering continuous or repeatable measurements without the need for blood drawing.

Over the past two decades, optical cytometry methods have emerged as promising candidates for non-invasive, label-free blood-cell detection, leveraging elastic light scattering [1], [2], [3], [4], absorption [5], [6], or Raman scattering [7] to differentiate circulating cells. The use of elastic scattering is especially appealing due to its relative simplicity and the distinct scattering properties of different blood cells [8]. In addition, scattering-based techniques such as spectrally encoded flow cytometry can visualize the morphology of circulating cells, which may be used for their identification [9], [10]. Nonetheless, despite progress in the field, purely optical methods have yet to demonstrate full *in vivo* classification of circulating blood cells.

Optoacoustic (also known as photoacoustic) flow cytometry can potentially supplement optical methods to improve their ability to perform complete blood counts. In contrast to elastic light scattering, which occurs for all blood cells, detectable optoacoustic signals are generated only by the most optically absorbing cells, which most often are the red blood cells, thus adding a layer of differentiation. Specifically, when blood vessels are illuminated with visible or near-infrared pulses, the rapid light absorption and thermal expansion of the red blood cells lead to the generation of short acoustic pulses, which may be detected by ultrasound transducers. A key application of optoacoustic flow cytometry is detecting circulating tumor cells, which has been demonstrated in the diffusion regime of light using an ultrasound transducer focused on the blood vessel of interest [11].

While acoustic focusing possesses the advantage of deep-tissue capabilities, optical focusing may be preferable for integration with optical methods, which operate in the ballistic regime, since its superior resolution enables identifying individual RBCs. Previous works have exploited this higher resolution for capillary imaging in a configuration known as optical-resolution photoacoustic microscopy (OR-PAM) [12]. By employing rapid scanning of the focused laser beam, real-time imaging rates have been obtained, enabling tracking of individual RBCs [13], [15] and initial clinical microvascular imaging in the oral cavity [14] and fingernail bed [43]. One of the limitations of such systems is their reliance on mechanical scanning and small water cavities to assure acoustic coupling between the tissue and ultrasound transducer, leading to bulky apparatus that is challenging to integrate with existing optical systems. An additional limitation is the traditional reliance of OR-PAM systems on opaque piezoelectric transducers, which requires specialized solutions to combine the optical and acoustic paths, limiting the use of high-magnification optical objectives. However, since the photoacoustic approach is limited to RBCs, there is a need for its integration with high-resolution microscopy capable of identifying other blood cells based on their morphology, namely platelets and white blood cells. We also note that the clinical demonstration of OR-PAM systems has not been performed for counting RBCs in a fixed position, a procedure more sensitive to motion than imaging.

In recent years, transparent ultrasound detectors have emerged as a promising approach for integrating optoacoustic imaging and sensing with optical methods, enabling one to perform the illumination through the ultrasound detector and integrating high-magnification objective lenses [16]. Originally, transparent ultrasound detectors were based on optical-resonator platforms, such as the free-space Fabry Perot [17], polymer micro-rings [18], [19], [20], pi-phase shifted Bragg gratings [21] and fiber-based Fabry-Perots [22], which measure acoustic pressure via optically induced resonance shifts arising from a combination of photo-elastic effects and mechanical deformation. Recent studies have demonstrated high-sensitivity and broadband performance using optical resonators such as micro-ring sensors [18], [23]. In the case of polymer micro-rings with high Q-factors, the miniaturization is typically limited to 60 µm to avoid radiation losses, leading to a tradeoff between the acoustic bandwidth and acceptance angle [23], [46]. Nonetheless, optimization of micro-ring geometry can lead to detection field of views of 600 µm, which is often sufficient for microscopy applications [20]. More recently, new transparent platforms for piezoelectric transducers have been developed as an alternative to optical detectors that additionally enable pulse-echo ultrasound [24], [25], [26], [27]. In the specific case of OR-PAM, the most common transparent configurations involved co-axial focusing of both the optical excitation and acoustic detection to maximize sensitivity. However, co-aligning the optical and acoustic paths leads to system complexities that may pose a challenge in demanding clinical applications.

In recent years, an alternative optical technology for ultrasound detection has been developed, known as the silicon-photonics acoustic detector (SPADE), which is based on a silicon [28], [29] or silicon-nitride [30] resonator coated with a transparent elastomer. By leveraging the advanced technological capabilities of silicon photonics, the SPADE platform has the potential to achieve a compact device geometry and compatibility with scalable fabrication processes, offering potential benefits for system integration and future translation. While originally developed for optoacoustic tomography [28], SPADE has also been demonstrated for OR-PAM [31].

In the case of OR-PAM, SPADE enables offset-detection geometries that allow scanning the optical beam away from the detector without mechanical scanning in the acoustic path [31]. To maximize the acceptance angle of SPADE, it is desirable to use π-phase shifted Bragg grating (π-BG) as the resonator, which leads to a sensor width on the scale of 1 µm and, accordingly, to semi-isotropic response in that direction for the entire measurement bandwidth [32].

In this work, we have developed a novel optoacoustic flow cytometry system for detecting RBCs in capillaries that are sufficiently superficial to enable optical focusing. The system is based on a new SPADE design that is transparent at visible wavelength, enabling its integration with a reflection-based microscopy setup, in which the focused illumination is transmitted through the detector. The system is integrated with a widefield optical microscope that can visualize the flow of RBCs in superficial capillaries through their absorption of back-scattered light. In contrast to OR-PAM systems, the illumination is not continuously scanned to image the RBCs in the capillaries but is rather tuned to a specific spot on one of the capillaries, guided by the microscope image, and measures the signal fluctuations at that spot. The SPADE design was based on a π-BG with effective dimensions of 1 × 82 µm (W×L), leading to acceptance angles of ±50° and ±7°, respectively, over bandwidth, which exceeds 140 MHz (Supplementary Fig. S3). Thus, this design achieves generally comparable angular sensitivity to polymer micro-ring resonators [23], [46] on its length axis, while offering semi-isotropic sensitivity on the width axis. The wide acceptance angle of SPADE along the width axis enables proper detection even when the detector is positioned at an offset from the focused beam. Importantly, this tolerance to imperfect alignment, combined with optical guidance, facilitates practical operation in dynamic measurement environments where precise co-localization is difficult to maintain.

The system was designed to perform optoacoustic flow cytometry in the oral mucosa of the lip. Accordingly, a custom-made lip holder was constructed to prevent motion during system operation, allowing monitoring of RBCs at a fixed location. The holder was deliberately designed with user comfort in mind, enabling stable positioning of the lip without discomfort or pain during measurements. In addition, the locking system between the interrogation setup and the SPADE resonator was improved to overcome drifts associated with the temperature and pressure increase due to the contact with the lip (Supplementary Note 5). The system was successfully demonstrated on a healthy volunteer at eight different positions, showing significant fluctuations in the optoacoustic signal over a timescale of six seconds.

## 2 Materials and methods

### 2.1 SPADE

**Fig. 1:**
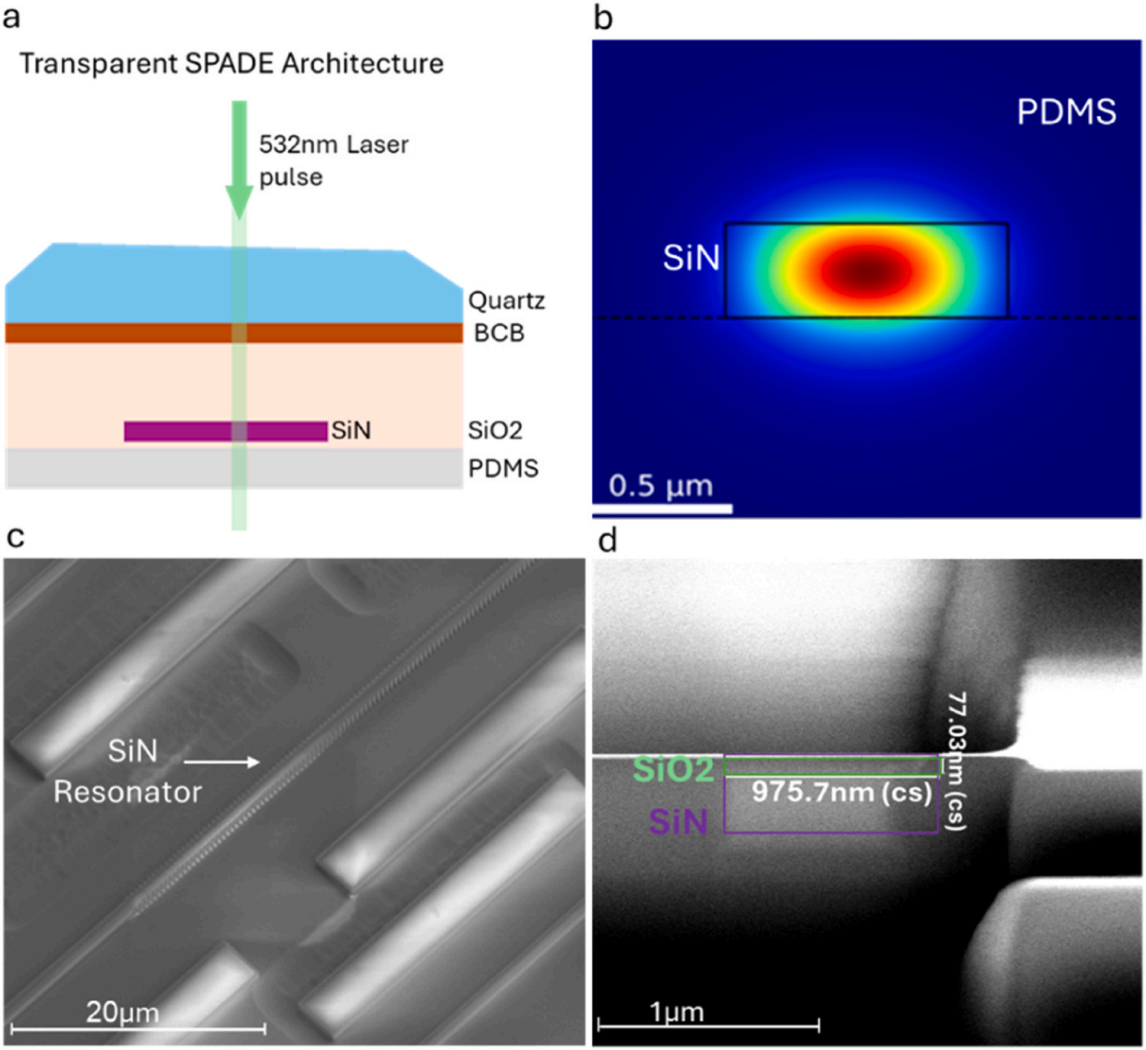
Structural characterization of the transparent SPADE resonator. (a) Illustration of the layer structure of the transparent SPADE. (b) Simulated optical mode distribution of the resonator, demonstrating strong confinement within the SiN core, with a portion of the optical field extending into the residual SiO₂ layer and the surrounding PDMS cladding. (c) Focused ion beam (FIB) image of the resonator region following controlled over-etching, in which the SiO₂ above the SiN resonator was fully removed to expose the resonator geometry. (d) High-magnification FIB image of the transparent SPADE in its final device cross-section, confirming the presence of a thin residual SiO₂ layer above the SiN waveguide core. The SiN core and the residual SiO₂ layer are explicitly labeled.

The fabrication process and sensing performance of SPADE were described in previous works [28], [29], [30]. Briefly, the detector is based on an optical resonator fabricated in a silicon nitride (SiN) platform (LGT-MPW-AN350, Ligentec), where ultrasound waves induce a shift in the optical resonance through their interaction with the optical mode as the acoustic field propagates through the surrounding medium. The cross section of the transparent SPADE used in this work is shown in Fig. 1a. The structure includes a SiN waveguide core with a cross-section of 350 nm (height) by 1 µm (width) that is partially buried inside a silicon oxide (SiO₂) cladding. The SiO₂ cladding extends 77 nm above the core’s top surface and is coated by polydimethylsiloxane (PDMS), which partially overlaps with the evanescent portion of the optical mode (Fig. 1b), to enhance the sensitivity [28]. The structure is supported by a quartz substrate (Quartz Plate OD25.4 mm*T0.1 mm, JGS1, MICQ) bonded to the cladding by benzocyclobutene (BCB) adhesive (Cyclotene 3022–57). The substrate-transfer procedure, in which the silicon-photonics chip’s original silicon substrate was replaced by quartz is described in Supplementary Note 1. Fig. 1c shows a top view image of the SiN resonator, when partially exposed, and Fig. 1d shows the device’s cross section when the core is fully embedded in the silica cladding; both images were acquired using a focused ion beam (FIB) system.

Two polarization-maintaining fibers are horizontally bonded to the grating couplers, with fiber end-faces polished at a 49° angle and coated with a 150 nm gold layer (electron-beam PVD, BAK−501A, Evatec AG, Switzerland) to redirect the emitted light vertically into the chip, even when the device is submerged in water. Light coupling between the fibers and the transverse electric (TE) mode of the SiN core is enabled by a double-apodized design, used in previous works [29], [30], [31]. Before the substrate transfer to the quartz layer, the SPADE device exhibited a fiber-to-fiber insertion loss of 15 dB, measured at a wavelength of 1555 nm, and a quality factor (Q-factor) of approximately 6 × 10^5^ for this central transmission resonance. After the transfer, the insertion loss increased to 19 dB since grating couplers were originally designed to exploit the reflection from the silicon-silica interface, which was lacking in the transparent design. In addition, the Q-factor in the transparent device was reduced to 2.5 × 10^5^, which may be explained by structural non-uniformities introduced by the additional etching steps and internal stresses in the device due to the bonding process.

### 2.2 Setup for phantom measurements

#### 2.2.1 Optoacoustic system

**Fig. 2:**
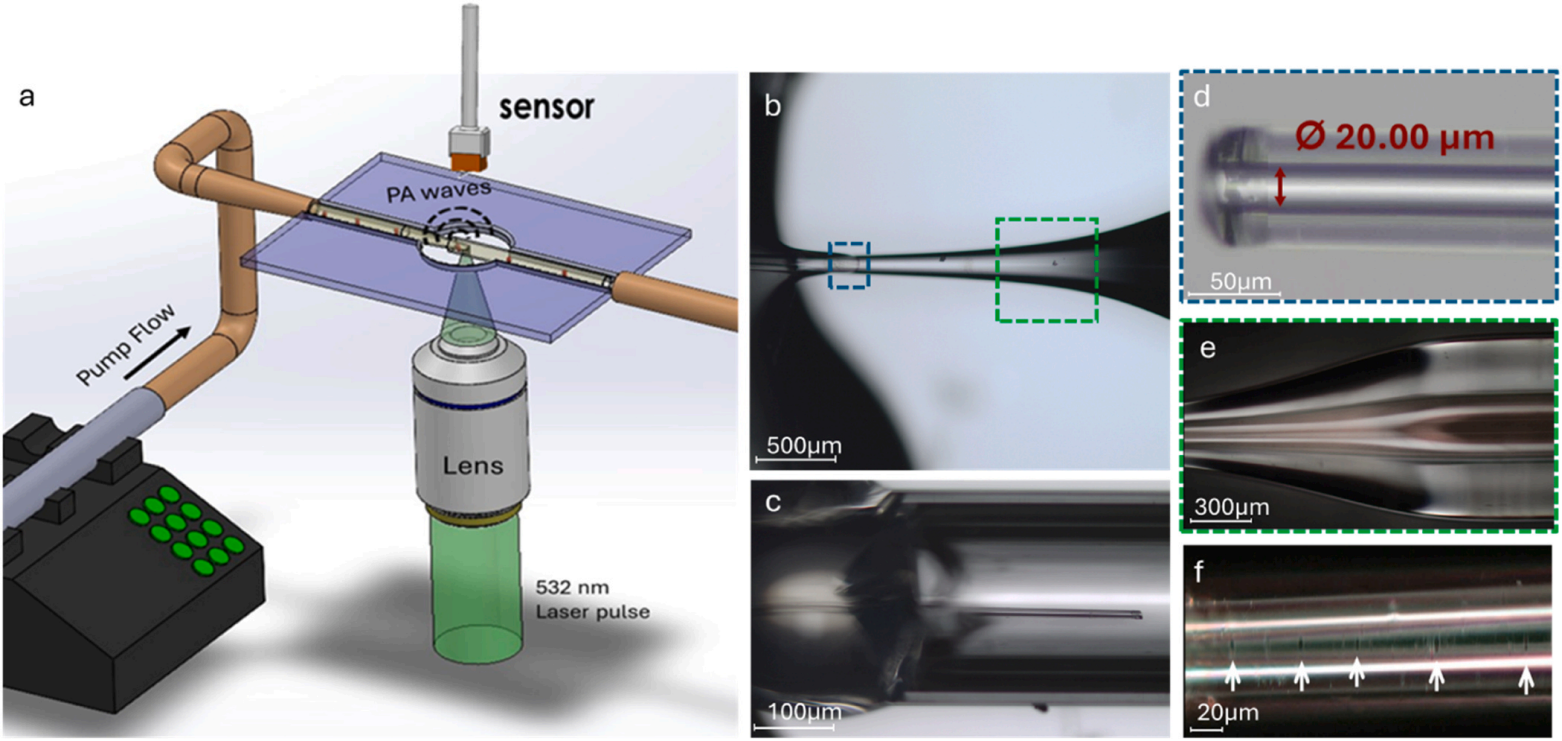
Microfluidic phantom for single-cell RBC flow. (a) Schematic of the optoacoustic excitation configuration, showing the focused 532 nm beam intersecting the tapered capillary. (b) Overview of the assembled phantom, where the tapered capillary is connected to a wider outlet tube leading to a collection reservoir. (c) Optical microscopy image of the taper region and the epoxy interface forming a continuous flow path. The blue square indicates the optoacoustic measurement region, and the green square indicates the tapering region. (d) Inner diameter at the narrowest section of the taper (≈20 µm), corresponding to the optoacoustic measurement location. (e) Optical image highlighting the taper geometry and gradual reduction in capillary diameter. (f) Enlarged view of the measurement region highlighted in (d), showing individual RBCs flowing through the capillary in a near single-file regime.

Optoacoustic excitation was performed using an Nd:YAG pulsed laser operating at a wavelength of 532 nm (Optogama, 'WAVEGUARD'), with a pulse width of 1 ns, a repetition rate of 1 kHz, and a maximum pulse energy of 80 µJ (“532 nm Laser”, Fig. 2b). The laser beam was attenuated, directed by two mirrors, and then coupled via an aspheric lens into a single-mode fiber with a core diameter of 3.2 μm (P1–460B-FC−1, Thorlabs). The average pulse energy at the fiber output was approximately 300 nJ. The beam was collimated and subsequently focused using a lens assembly comprising a plano-convex and an aspheric condenser lens, followed by an objective lens with a numerical aperture (NA) of 0.3 (CFI Plan Fluor, Nikon), resulting in a lateral focal width of 1.3 µm.

#### 2.2.2 Microfluidic chip

The microfluidic phantom used for controlled RBC flow experiments is shown in Fig. 2. A glass capillary (outer diameter [O.D.] 1 mm, inner diameter [I.D.] 0.3 mm; Sutter Instrument) was tapered using a micropipette puller (P−2000, Sutter Instrument) to form a narrow inlet channel that hydrodynamically guides the RBC flow into a smaller cross-section (Fig. 2b-c). This tapering is designed to gradually confine the flow so that RBCs enter the laser interrogation region individually rather than as clusters. As shown in Fig. 2b, the tapered capillary empties into a wider cylindrical glass tube (inner diameter 0.7 mm; Sutter Instrument), which serves as the outlet channel and is connected at its distal end to a collection reservoir where RBCs accumulate after passing through the measurement region. The channels were sealed with epoxy to form a continuous flow path (Fig. 2c).

The thinnest portion of the tapered capillary, located near the epoxy bonding point, was used as the measurement site, as highlighted in blue square in Fig. 2c. The inner diameter in this region was approximately 20 µm, as measured with an optical microscope (Eclipse E200, Nikon Instruments Inc.) and shown in Fig. 2d. A focused 532 nm laser pulse was directed across the capillary to excite individual RBCs flowing through this narrow segment (Fig. 2a). The flow rate was controlled using a syringe pump (Harvard Elite 11, Harvard Apparatus) to ensure stable and repeatable single-cell passage through the interrogation zone. Details of RBC preparation are provided in Supplementary Note 2. Representative RBC trajectories within the capillary are shown in Fig. 2f.

### 2.3 Setup for in vivo measurements

**Fig. 3:**
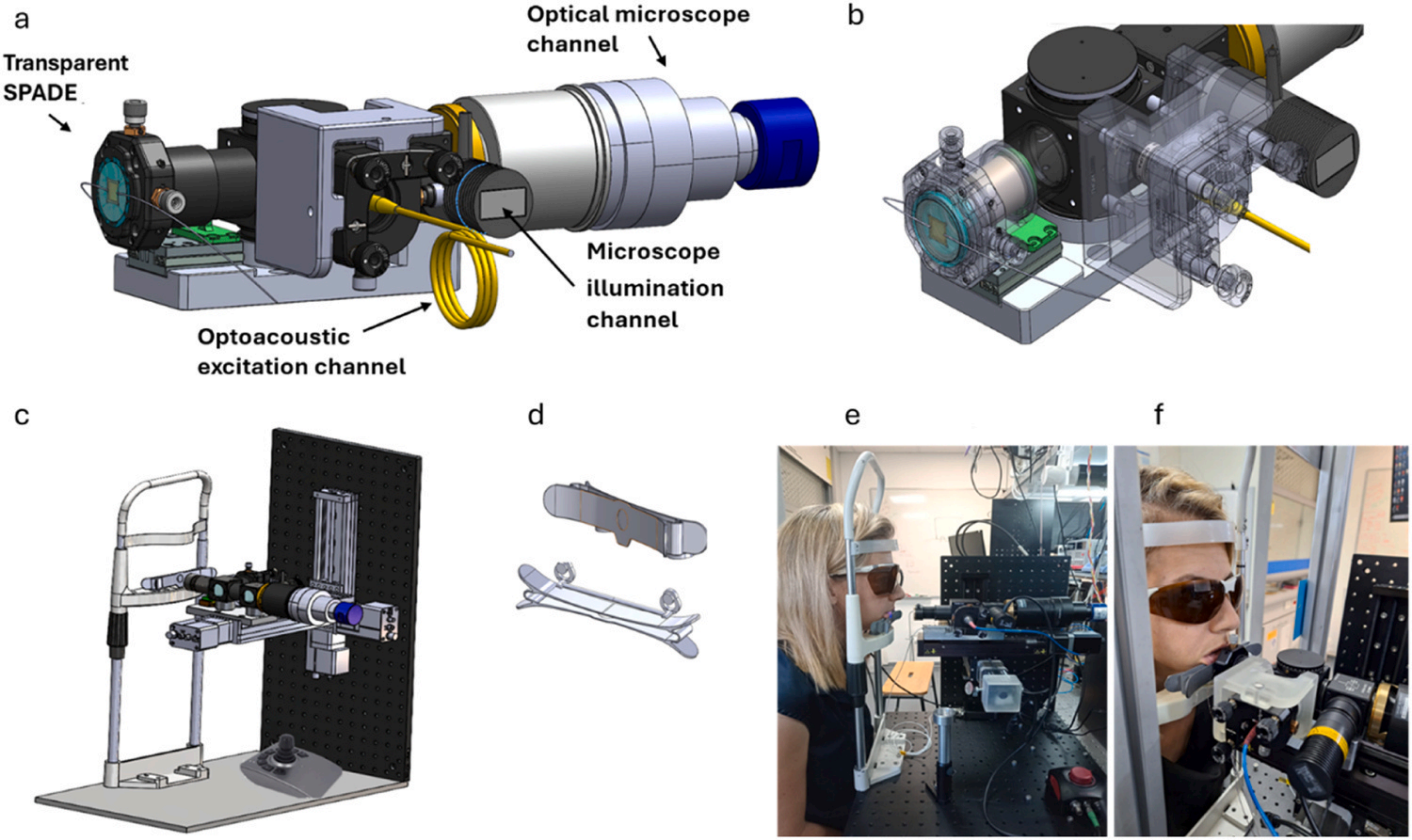
Integrated *in vivo* measurement setup. (a) CAD rendering of the compact and portable device showing the optoacoustic excitation channel, optical microscope imaging channel, and illumination path, with the transparent SPADE sensor positioned at the front of the system. (b) Semi-transparent view illustrating the beam steering mechanism (pan and tilt adjustment), together with the mechanical linear stage and objective configuration used for precise positioning of the optical focus. (c) Complete experimental setup mounted on a three-axis translation stage, enabling accurate positioning of the device during measurements. (d) Mechanical design of the lip holder used to stabilize the subject during measurements. (e) Photograph of the system during alignment and calibration, demonstrating the compact and integrated configuration prior to measurements. (f) Representative *in vivo* measurement configuration showing the device positioned in front of a volunteer’s lip for capillary imaging.

The integrated *in vivo* measurement setup is illustrated in Fig. 3. In total, measurements were performed at more than eight superficial inner lip capillaries in 3 healthy volunteers. All procedures adhered to institutional guidelines and the principles of the Declaration of Helsinki. The experimental protocol for lip-capillary measurements was reviewed and approved by the local Helsinki Committee for human subject research (approval # 202–2024), and written informed consent was obtained from all participants prior to the experiments.

For the *in vivo* experiments, the measurement setup consisted of three integrated channels combined into a single portable device. These channels included: (1) the optoacoustic excitation channel, used to deliver the focused pulsed laser beam to the tissue; (2) the optical microscope imaging channel, used for real-time visual alignment of the detector with superficial inner lip capillaries, and (3) the illumination channel of the optical microscope, which provided uniform lighting for clear visualization of the fiber resonators than the capillary network. The transparent SPADE sensor was positioned at the front of the device, as shown in Fig. 3a. The sensor was mounted on an XY mechanical stage (CXY1, Thorlabs), which allowed fine lateral adjustments to calibrate its position and to bring the relevant waveguide resonant sensing area as close as possible to the targeted capillary during the experiment. The entire assembly was mounted on a three-axis translation stage (Fig. 3c) controlled by dedicated software, enabling immediate and precise positioning of the device using the computer’s arrow keys in a manner similar to operating a joystick.

#### 2.3.1 Optoacoustic excitation channel

The optoacoustic laser-to-fiber coupling setup was similar to the configuration used for the microfluidic phantom measurements described in 2.2, with the addition of protective endcaps (Customize End cup cable, FAST laser group) mounted on both ends of the single-mode fiber to prevent damage or burning at the fiber facets during operation. The average pulse energy at the fiber output was approximately 800 nJ. The laser intensity complied with the IEC 60825–1 safety standard and was approved by an external laser safety expert.

Additional details regarding laser power at the capillary are provided in Supplementary Note 3, and safety considerations are provided in Supplementary Note 4. The emitted beam was first collimated using a fiber collimator (CFC11P-A, Thorlabs) passed via dichroic beam splitter (T425lpxr,Chroma) and then focused with an objective lens with a numerical aperture (NA) of 0.25 (Plan Achromatic, Olympus). Due to the beam diameter at the entrance of the objective, the effective NA was reduced to 0.05, resulting in a focal spot size lower than 4 µm. The mechanical holder of the single-mode fiber and its collimator (K5X1, Thorlabs) enabled pan and tilt adjustment, allowing precise control of the laser spot location and accurate calibration of its position anywhere within the optical microscope’s field of view depending on the chosen target capillary location. The microscope objective was mounted on a miniature motorized z-axis stage (XLS1, Xeryon), allowing precise adjustment of the focal position. This enabled fine tuning of the laser focus to match the depth of the specific capillary selected for each measurement set (Fig. 3b).

#### 2.3.2 Optical microscope channel

In addition to the optoacoustic excitation channel, an optical microscope channel was designed to provide real-time visualization of the inner lip capillaries for alignment purposes. For each experiment, precise guidance of the laser beam to the target spatial location and accurate adjustment of the laser focus to the depth of the selected capillary were required. The optical microscope channel comprised a C-mount imaging assembly (Edmund Optics), and a 180 mm tube lens (86–835, Edmund Optics). A camera (11–503, Edmund Optics) with a field of view of 1° was selected to provide a sufficiently wide imaging area, enabling reliable guidance of the sensor waveguide resonance toward the targeted capillary.

#### 2.3.3 Optical microscope illumination channel

The illumination channel of the optical microscope provided uniform lighting for visualization of the inner lip capillaries. Illumination was generated using a light-emitting diode source (M430L5, Thorlabs) operating at a central wavelength of 430 nm. The emitted light was shaped and directed using an aspheric condenser lens (ACL2520U–A, Thorlabs). Mechanical lens tubes (SM1 series, Thorlabs) were used to mount and align the optical components, allowing precise calibration of the condenser lens position to achieve collimated illumination at the sample plane. This configuration ensured wide homogeneous illumination across the microscope field of view during in-vivo measurements.

#### 2.3.4 Lip and face holder for human volunteers

A dedicated lip holder was designed to stabilize the measurement region during the *in vivo* experiments. The holder allowed the subject’s lips to be positioned within the device, with a circular opening at its center marking the designated measurement area. To ensure comfort and mechanical compliance, the holder was fabricated using three-dimensional printing with Nylon−12 material. Two supporting arms extended from the lip holder, enabling rigid attachment to a facial support frame. This configuration provided mechanical stability and minimized motion of the lips during measurements (Fig. 3d). In addition, a separate facial support structure was designed to reduce the volunteers’ involuntary head and facial movements, further stabilizing the experimental setup throughout data acquisition (Fig. 3e-f).

### 2.4 Interrogation system

The optical interrogation system for SPADE is based on monitoring the refractive-index modulation caused by the impinging acoustic waves. In this study, we employed the phase-monitoring technique, which enables shot-noise detection for high-Q resonators [33]. This technique involves tuning a continuous laser to the maximum of the transmission resonance, where the resonator’s phase response is linear. Accordingly, the optical signal resonance output experiences a phase modulation that is proportional to the acoustically induced wavelength modulation. To detect this phase modulation, we utilized an interrogation system based on a Mach-Zehnder interferometer (MZI) as described in previous works [28], [29], [30], [31]. The output of a tunable continuous-wave (CW) laser was split between a sensing arm with the SPADE chip and a reference arm with a piezoelectric fiber stretcher (OPTIPHASE, PZ3) and optical delay-line (OZ optics, ODL−100). The optical path difference between the two arms was minimized to reduce the effect of laser phase noise, and the arms’ outputs were recombined and delivered to a balanced photodetector. The MZI was stabilized to quadrature by the fiber stretcher, where the differential signal is zero, using a feedback circuit with a bandwidth of 3 kHz [29]. When the wavelength of the resonator was acoustically modulated at frequencies above 3 kHz, the induced phase shift was not compensated by feedback circuit, leading to a modulation in the output voltage signal which was recorded by a digitizer (PicoScope5444D, Pico technology).

## 3 Results

### 3.1 Optoacoustic performance of the transparent SPADE

**Fig. 4:**
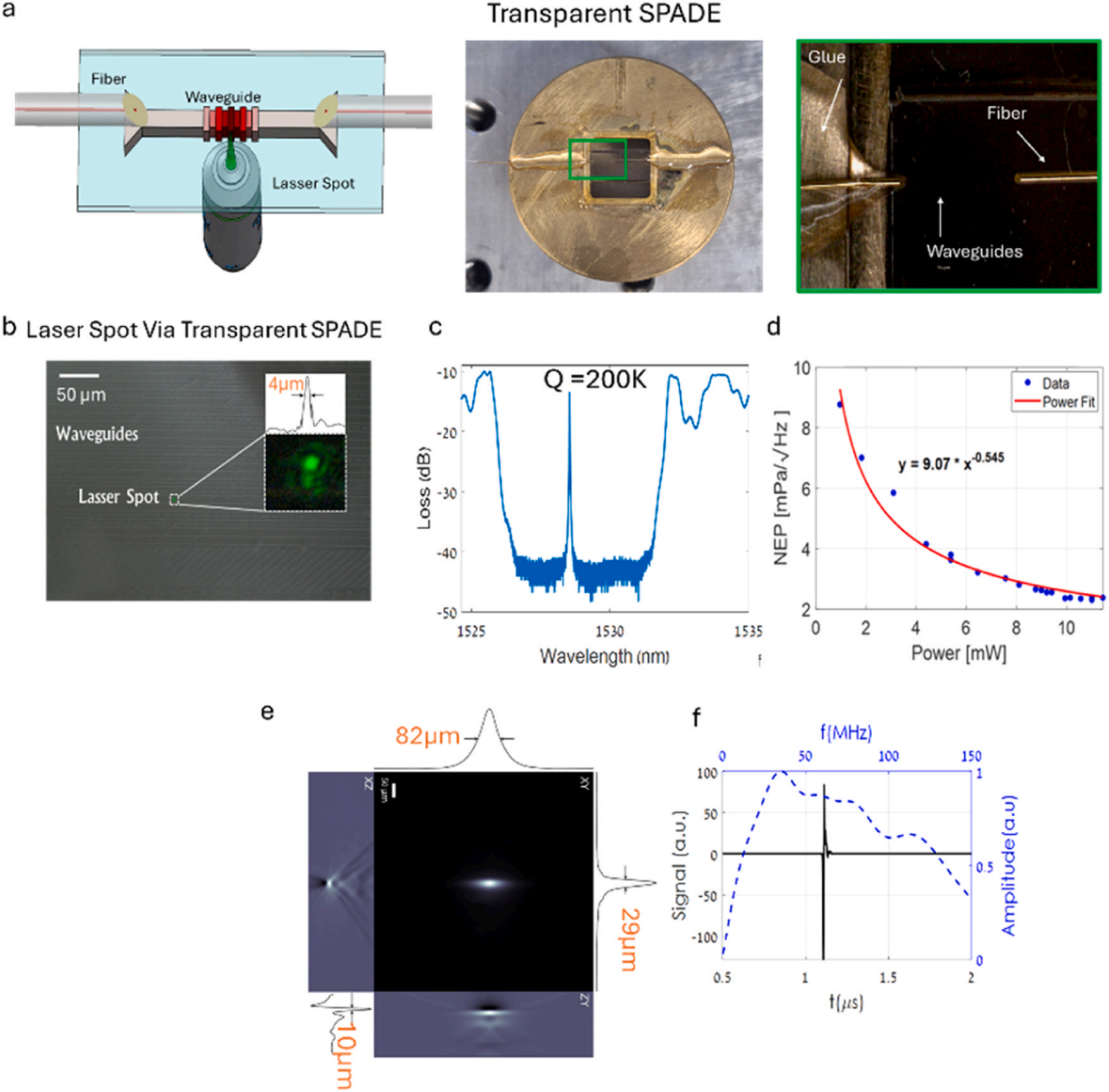
Optical and optoacoustic characterization of the transparent SPADE. (a) Schematic illustration (left) and photographs (center and right) of the transparent SPADE. The illustration shows how laser illumination path passes through the sensor. The photograph on the right shows a magnification of the region contained in the green square in the central image. (b) Optical microscope image of the laser spot transmitted through the transparent SPADE and focused onto the waveguide region. The inset shows the measured intensity profile of the laser spot, with a focal diameter of approximately 4 µm. (c) Optical transmission spectrum of the transparent SPADE resonator, showing a resonance corresponding to a quality factor of approximately Q = 2 × 10⁵ near 1530 nm. (d) Measured noise-equivalent pressure (NEP) of the transparent SPADE as a function of optical interrogation power, demonstrating improved sensitivity with increasing optical power. (e) Spatial optoacoustic response of the transparent SPADE obtained using a localized optoacoustic point source positioned 0.8 mm from the sensor, demonstrating sensitivity over a wide acceptance angle. (f) Representative optoacoustic time-domain signal generated by a black ink absorber (edding 750) and detected by the transparent SPADE (black line). The corresponding frequency spectrum (blue dashed) demonstrates broadband acoustic detection extending beyond 100 MHz.

To comprehensively characterize the performance of the transparent SPADE, a series of optical and optoacoustic measurements were conducted, as summarized in Fig. 4. Fig. 4a shows the fabricated transparent SPADE device together with a schematic illustration of the optical excitation geometry, demonstrating that the pulsed laser illumination is transmitted through the sensor and focused on close proximity to the waveguide region. Preservation of optical focusing through the detector was experimentally verified (Fig. 4b), where the transmitted focal spot was imaged at the waveguide plane. The measured spot profile indicates a focal diameter below 4 µm, confirming that tight optical focusing is maintained despite propagation through the multilayer sensor stack.

The optical resonator characteristics of the transparent SPADE are presented in Fig. 4c. A resonance quality factor of approximately 2 × 10⁵ was measured near 1530 nm, indicating that the additional fabrication steps required to achieve optical transparency did not significantly degrade the resonator performance.

Acoustic sensitivity was quantified by measuring the noise-equivalent pressure (NEP) as a function of the optical interrogation power (Fig. 4d). The NEP decreased from approximately 8.5 mPa/Hz^1/2^ at 1 dB to 2.3 mPa/Hz^1/2^ at 10 dB, following a square-root dependence on the power, expected for shot-noise-limited detection [33]. The reported NEP values are bandwidth normalized. Specifically, the NEP was calculated by dividing the mean standard deviation of the background voltage noise by the detector responsivity and by the square root of the detection bandwidth. For application-level interpretation, the bandwidth-normalized NEP of 2.3 mPa/Hz¹ ᐟ² corresponds to an integrated NEP of approximately 20.6 Pa over the effective 10–90 MHz bandwidth used in the analysis.

The spatial optoacoustic response of the transparent SPADE was characterized using an ink absorber (edding 750, Edding) acting as an optoacoustic point source positioned 0.8 mm from the sensor (Fig. 4e). The optoacoustic reconstruction revealed a line-shaped point-spread function (PSF) in the lateral plane, reflecting the elongated geometry of the sensing region. The lateral full-width-at-half-maximum (FWHM) was 82 µm along the long axis of the sensing region and 29 µm along the orthogonal lateral direction, while the axial FWHM along the acoustic propagation direction was approximately 10 µm.

Finally, the temporal and spectral responses were evaluated using the ink absorber (Fig. 4f). The time-domain signal exhibited the expected sharp bipolar optoacoustic transient, and the corresponding frequency spectrum confirmed broadband acoustic detection extending beyond 100 MHz. Together, these results demonstrate that the transparent SPADE combines high optical quality, preserved optical focusing through the detector, low noise-equivalent pressure, and broadband acoustic sensitivity, making it well suited for micron-scale optoacoustic detection. The angular response of the transparent SPADE used in the present study was additionally characterized and is provided in Supplementary Fig. S3, confirming a broad acoustic response over the measured angular range.

### 3.2 Phantom measurement results

**Fig. 5:**
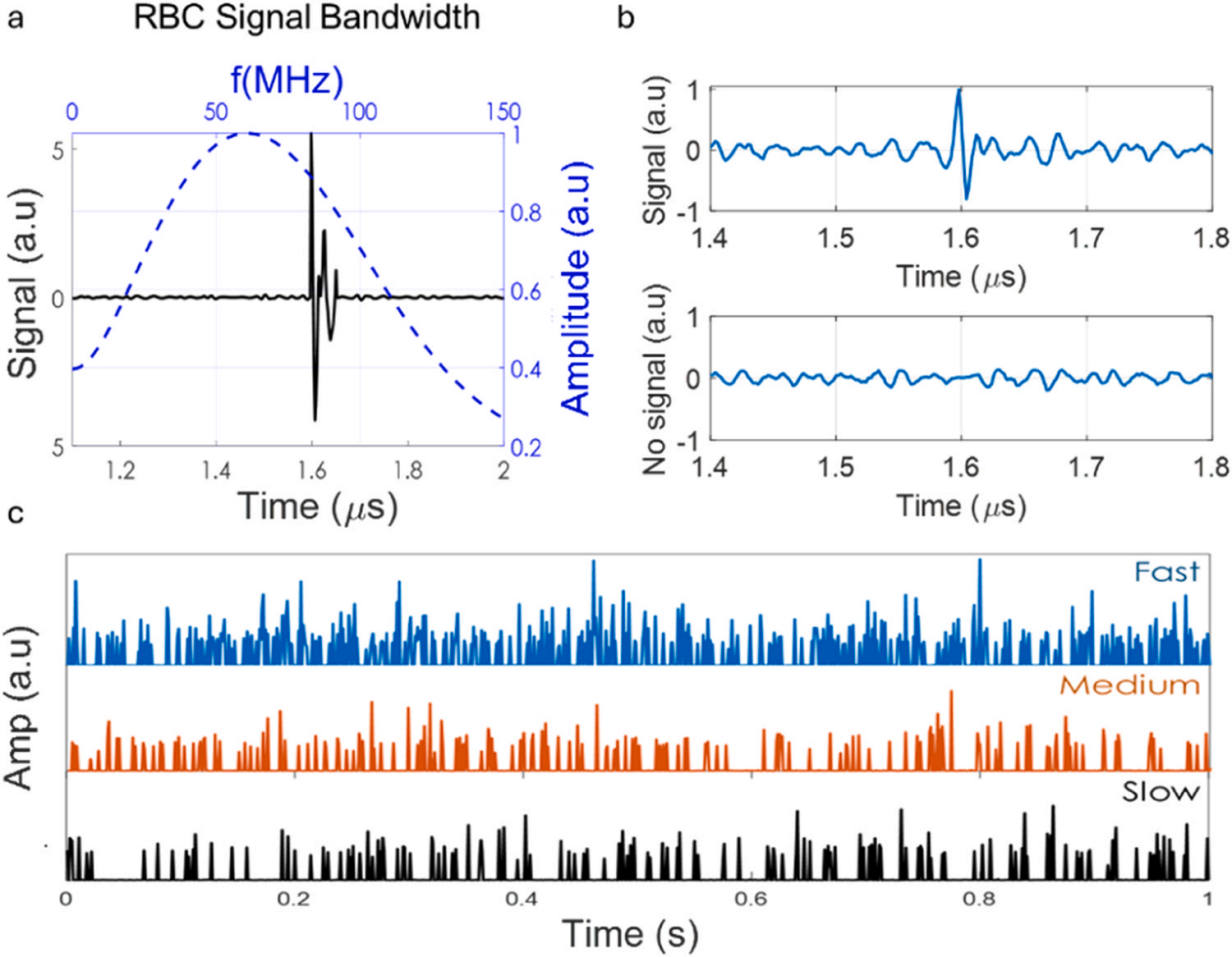
Phantom measurement results and red blood cell flow characterization. (a) Time-domain optoacoustic signal generated by an individual red blood cell and its corresponding frequency spectrum, demonstrating broadband acoustic content extending beyond 100 MHz. (b) Representative optoacoustic signal attributed to a single red blood cell (top) and a background trace acquired in the absence of red blood cells (bottom), illustrating the achieved signal-to-noise contrast. (c) Time traces of detected optoacoustic events acquired under different nominal flow conditions (low, medium, and high), showing an increased temporal density of events with increasing flow setting.

Phantom experiments were performed to evaluate the ability of the system to detect RBC-related optoacoustic events under controlled flow conditions. Representative optoacoustic signals acquired from the microfluidic phantom are shown in Fig. 5. Transient responses attributed to individual RBC passages were clearly distinguishable from background noise across a range of nominal flow settings.

For quantitative analysis, the raw optoacoustic signals were processed using a detection pipeline designed to preserve transient waveform features relevant for event identification. The DC offset was removed, and a temporal window corresponding to the expected signal region of interest was defined around each candidate event. The signals were bandpass filtered between 10 and 90 MHz using a fourth-order Butterworth filter to suppress low-frequency drift and out-of-band noise while retaining the broadband content of RBC-generated transients. A Savitzky–Golay filter (third-order polynomial) was then applied to reduce residual noise without distorting waveform morphology. Additional smoothing, implemented as a moving-average filter with a window length of 31 samples, was applied only for visualization purposes and only to background regions outside the signal window. This display-only processing was not used for event detection and did not affect the noise statistics, detection threshold, or any quantitative analysis.

RBC-related events were identified based on the peak-to-peak amplitude of the processed optoacoustic transients. A noise-referenced threshold was applied, and only events exceeding three times the standard deviation of the background noise were classified as RBC-related events. To avoid multiple detections of the same cell, the temporal definition of an event and the minimum separation between consecutive events were chosen based on a relative estimate of the expected RBC transit time through the excitation volume. This estimate accounts for the characteristic RBC size, the nominal flow conditions imposed by the syringe pump, the laser repetition rate, and the optical focal spot dimensions, without assuming precise knowledge of the local flow velocity at the measurement site. Consequently, the analysis focuses on relative trends in event occurrence as a function of flow conditions rather than on absolute cell counting.

Fig. 5a shows the temporal waveform and corresponding frequency spectrum of a representative optoacoustic event, demonstrating broadband acoustic content extending beyond 100 MHz, consistent with micron-scale absorbers such as individual RBCs and the wide detection bandwidth of the transparent SPADE sensor. Event trains recorded at different nominal flow settings (Fig. 5c) exhibit an increased temporal density of detected events with increasing flow rate, while the waveform morphology remains consistent, indicating stable single-cell sensitivity across flow regimes. A comparison between the optoacoustic results and those obtained with an optical setup is shown in Supplementary Fig. S2.

Variations in optoacoustic signal amplitude between individual events were observed and are attributed to differences in cell orientation and spatial position within the laser focal volume. In addition, since the microfluidic channel width exceeds the RBC diameter, small lateral or axial offsets within the focal region can lead to local fluence variations. Such variability is intrinsic to single-cell flow measurements and does not compromise the reliable detection of individual RBC-related events.

### 3.3 In vivo measurements results

**Fig. 6:**
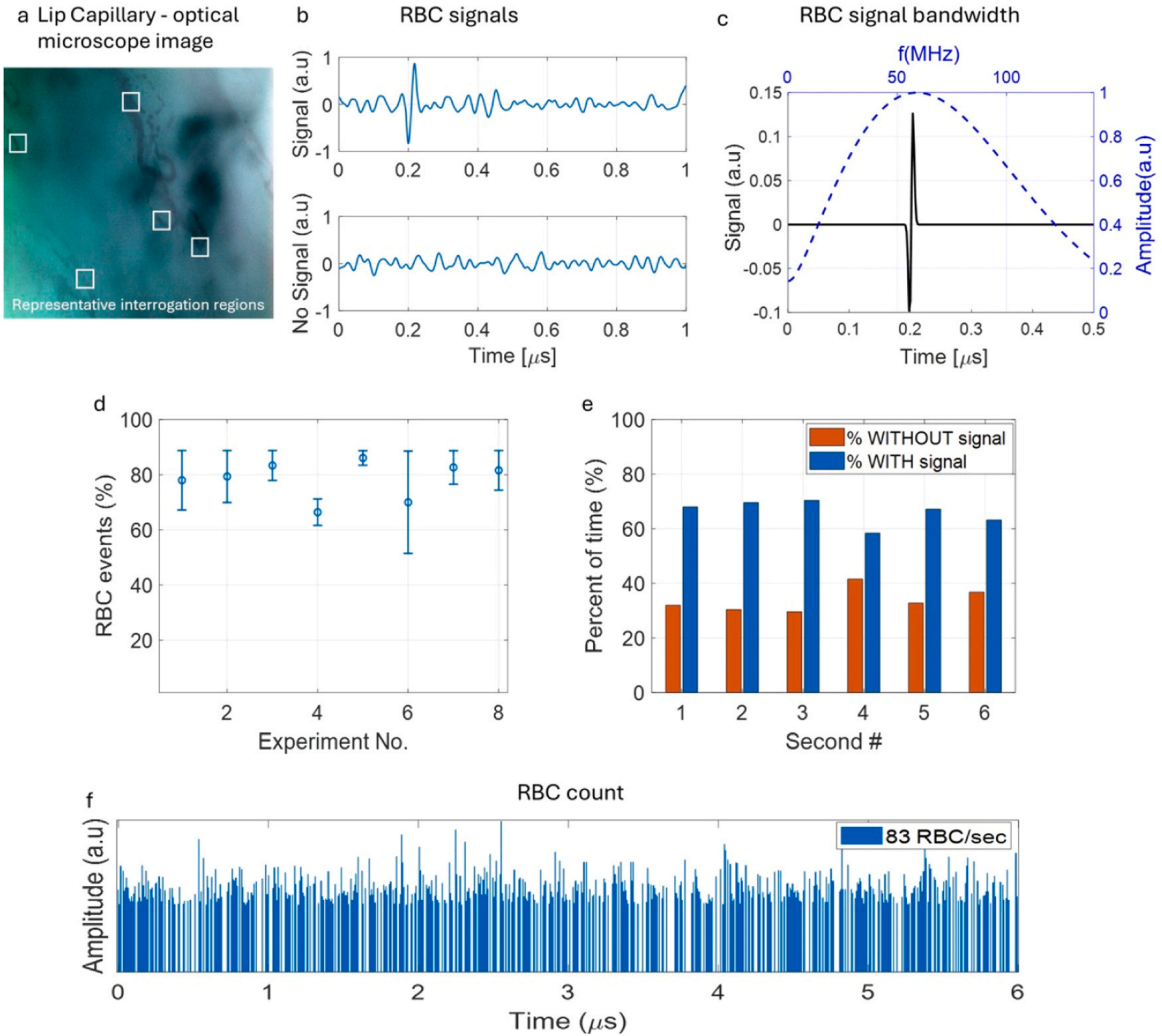
Representative *in vivo* measurements and RBC event analysis in superficial inner lip capillaries. (a) Optical microscope image of the lip capillary network used to guide alignment of the transparent SPADE sensing region and the excitation beam. Examples of representative optoacoustic interrogation regions are indicated in white squares. These regions feature the type of capillary morphology and visibility that were targeted when aligning the optical beam. (b) Representative time traces showing an optoacoustic transient attributed to a single RBC passage (top) and a no-event segment (bottom). (c) Time-domain optoacoustic waveform of a representative RBC event (black) and the corresponding frequency spectrum (blue dashed), demonstrating broadband content. (d) RBC event fraction (RBC-related events as a percentage of total detected events) obtained across multiple *in vivo* measurements. (e) Per-second segmentation of the recording in (f), showing the percentage of windows classified as “with signal” and “without signal,” highlighting temporal variability in capillary flow activity. (f) Continuous *in vivo* recording showing an RBC event train; the event rate in this example was approximately 83 RBC/s.

*In vivo* experiments were performed on superficial capillaries in the inner lip of healthy human volunteers using the transparent SPADE in an epi-illumination configuration. Real-time optical microscopy enabled precise alignment of the sensing region and the focused excitation beam to a selected capillary (2.3), after which optoacoustic signals were acquired from a fixed spatial position. To achieve high sensitivity, the optical beam was first aligned to the approximate position of the SPADE sensor and then translated along its width axis, where the wide angular sensitivity is achieved, until reaching the capillary. Representative measurements are shown in Fig. 6.

Distinct transient optoacoustic events were consistently detected and attributed to individual red blood cell (RBC) passages through the optical excitation volume, with clear separation from no-event segments (Fig. 6b). The corresponding time-domain waveforms and frequency spectra (Fig. 6c) exhibited broadband acoustic content consistent with micron-scale optical absorbers such as individual RBCs, consistent with the wide detection bandwidth of the transparent SPADE sensor.

To improve the signal-to-noise ratio while preserving the transient nature of single-cell signals, each detected event was analysed after averaging over a limited number of consecutive samples. The number of averages was chosen based on a physiologically motivated estimate of the RBC transit time through the excitation volume. Reported RBC velocities in human oral and labial mucosal capillaries are typically on the order of 0.5–1.5 mm/s [34], [35].

The effective interaction length along the flow direction was estimated as the sum of the laser focal spot size and the effective axial length of an RBC [36], [37] confined within a capillary, yielding a total interaction length of approximately 10 µm. Considering an acquisition sampling rate of 1 kHz, a modest averaging over eight consecutive samples was applied, providing noise reduction while remaining within the estimated single-cell transit duration and avoiding temporal smearing of adjacent events.

RBC event detection followed the same noise-referenced approach used in the phantom experiments. Events were identified based on their peak-to-peak amplitude, with a detection threshold set at three times the standard deviation of the background noise. To avoid multiple detections originating from a single cell, the temporal definition of an event and the minimum separation between consecutive events were chosen based on the estimated transit time, without assuming precise knowledge of the instantaneous local flow velocity within the capillary.

Across multiple measurement locations, a high fraction of detected events was consistently classified as RBC-related (Fig. 6d), indicating robust single-cell sensitivity under *in vivo* conditions. Continuous recordings revealed sustained RBC event trains over several seconds (Fig. 6e). The example shown corresponds to an event rate of approximately 83 RBC/s, while other capillary locations exhibited different rates, reflecting physiological variability in microvascular blood flow. Per-second segmentation of the same recording (Fig. 6f) demonstrated temporal fluctuations in the fraction of time windows containing detectable RBC-related events, consistent with natural variability in capillary flow as well as residual motion and physiological dynamics in microcirculation [38], [39], [40]. These observations further support the interpretation that the detected signals originate from individual RBC passages under *in vivo* conditions.

## 4 Discussion

In this work, we developed and demonstrated a novel optoacoustic flow cytometry system for probing superficial capillaries and detecting red blood cells in the inner lip of healthy human volunteers. The system combines focused optical excitation for generating optoacoustic signals from individual RBCs with a transparent silicon-photonics acoustic detector (SPADE) for signal detection. The transparency of SPADE provides two key advantages. First, it enables optoacoustic excitation to be delivered directly through the detector, eliminating the need for optical–acoustic beam combiners and thereby reducing system complexity. Second, it allows seamless integration of a widefield optical microscopy channel into the optoacoustic setup. This optical channel is essential for visual guidance, enabling accurate positioning of the excitation beam on superficial capillaries where discrete RBC flow can be directly observed. In addition, the wide acoustic acceptance angle of the SPADE relaxes the requirement for strict co-alignment between the optical excitation and acoustic detection, which is a key constraint in conventional optical-resolution optoacoustic systems. This enables a more flexible system geometry and facilitates stable measurements under practical *in vivo* conditions.

The SPADE employed in this study is based on a one-dimensional optical cavity formed in a silicon nitride waveguide, exhibiting a quality factor of approximately $2\times 10^{5}$. When combined with a low-noise interrogation scheme, this resulted in a noise-equivalent pressure (NEP) of approximately 2.3 mPa/√Hz. While the substrate-transfer process required to achieve optical transparency altered the acoustic response of the detector, likely due to internal reflections within the layered structure, the transparent SPADE nevertheless demonstrated a broadband acoustic response extending beyond 100 MHz.

Importantly, optoacoustic signals generated by individual RBCs exhibited spectral content spanning this full bandwidth, whereas background signals originating from bulk tissue were dominated by lower-frequency components. This spectral separation provides an inherent mechanism for distinguishing RBC-related events from background contributions. The effective sensitive region of the SPADE forms a short line segment with an approximate length of 82 µm and width 1 µm, respectively, enabling detection of optoacoustic sources even when the excitation beam is laterally offset from the detector. As a result, precise co-localization between the optical focus and the detector is not required for system operation.

The optoacoustic flow cytometry system was successfully evaluated in both microfluidic flow phantoms and *in vivo* measurements in the oral mucosa of the lip. In the phantom experiments, diluted RBC suspensions produced sparse flow conditions in which individual RBCs could be readily identified in the optical channel. Correspondingly, the optoacoustic measurements revealed an increase in the detected event rate with increasing flow. When the pump was turned off, leading to a diffusive-flow regime, the resulting optoacoustic event rate decayed over time, in agreement with the results obtained by an optical system under the same conditions.

In contrast to the phantom measurements, the *in vivo* measurements exhibited substantially higher RBC densities, with cells occupying most of the capillary length, as clearly observed in the widefield optical videos. Under these conditions, optoacoustic events were detected during the majority of the recording duration, resulting in high event occupancies. As illustrated in Fig. 6, measurements characterized by lower occupancies also exhibited less regular temporal behavior, reflected by increased variability in the detected signals. Optical recordings corroborated these findings, showing unsteady and intermittent flow patterns in capillaries with sparser RBC distributions.

Importantly, the stability of the detected RBC-related transients over extended *in vivo* acquisition periods indicates that the system can maintain reliable optoacoustic sensing under realistic measurement conditions. This capability is achieved despite the challenges associated with direct measurements in human tissue, including mechanical motion, physiological variability, thermal drift, and variations in optical and acoustic coupling, while maintaining fixed-position operation over extended acquisition periods. Our results demonstrate that the combination of wide-angle acoustic detection and integrated optical guidance effectively mitigates these effects, enabling continuous or repeated monitoring in human capillaries.

The current work represents a proof-of-concept study for the incorporation of optoacoustic flow cytometry into optical techniques for non-invasive blood count, and several improvements may be made in future studies for both the system and detector level. On the system level, it is clear that with current performance a true count of RBCs cannot be performed without correlating the optoacoustic data with optical images because of the irregular blood flow. For example, a slow RBC may create the same optoacoustic signals as two fast RBCs that flow in tandem, making them indistinguishable by optoacoustic flow cytometry. Therefore, it remains crucial to optimize the images generated by the optical channel. In our system, higher quality images could have been achieved by using well-known techniques that enhance the contrast between the RBCs and background, e.g., using an objective lens with a higher magnification, and improving the transparency of the SPADE. The optoacoustic system may be improved by using motorized mirrors to control the beam’s position, enabling automatic positioning of the beam onto the capillaries.

At the detector level, further improvements in SPADE sensitivity and bandwidth could extend the applicability of the technique to deeper capillaries. Specifically, higher sensitivity may be achieved through an improved substrate transfer procedure that does not deteriorate the resonator’s Q-factor. Increased acoustic bandwidth, particularly toward 200 MHz, may also enable discrimination between closely spaced RBCs traveling in tandem. At such frequencies, the optoacoustic spectral signature of an individual RBC becomes increasingly sensitive to cell size and orientation, potentially allowing identification of individual cells based on spectral features alone, without reliance on optical imaging. Although acoustic attenuation in tissue is expected to limit the usable bandwidth, frequencies approaching 200 MHz may remain accessible at depths on the order of 200 µm. Indeed, previous studies have reported attenuation values of approximately 3–4 dB over 100 µm propagation distances in kidney and muscle tissue at 200 MHz [41], [42], supporting the feasibility of high-frequency optoacoustic detection in superficial biological tissues.

Finally, we note that alternative ultrasound detection technologies may be used in future sensing systems. In case only an RBC count is required, conventional OR-PAM systems [43], [44], [45] that rely on opaque piezoelectric transducers may be used in imaging mode, thus obviating the need for optical guidance. However, this approach comes at the cost of higher complexity of the optoacoustic system, requiring fast scanning mirrors and beam combiners for the optical and acoustic paths. Moreover, this approach inherently limits integration with high-resolution microscopy due to the long propagation paths imposed by the beam combiners, which limit the use of high-magnification objectives. If a full blood count is desired, integration with optical microscopes is essential since OR-PAM is sensitive only to the RBCs, making transparent ultrasound transducer a potentially more favorable option.

The advantage of a transparent SPADE is its high acceptance angle, which may reach a typical half-maximum value of 5*0*°. While in this work the high acceptance angle was achieved only on the width axis, it may be achieved also on the length axis by using shorter designs [28]. Alternative transparent technologies may also be used in the same application, at the possible cost of a more elaborate positioning system or procedure. In the case of polymer micro-rings, fields of view of up to 0.6 mm have been demonstrated in OR-PAM system [20], which may be sufficient also for clinical RBC systems. Since the acceptance angle of micro-rings scales with the ratio of the acoustic wavelength and micro-ring diameter 47]], their field of view may be increased by operating in a lower bandwidth or using smaller resonators. However, using smaller micro-rings may limit the Q-factor, due to higher radiation losses [48], and reducing the bandwidth will lead to weaker signals. Nonetheless, polymer micro-rings have an inherent potential for higher sensitivity, which may ameliorate these effects, since effectively all the optical mode is contained in the polymer layer in which sensing occurs, whereas in SPADE the overlap with the sensing polymer layer is generally less than 20%.

## CRediT authorship contribution statement

**Amir Rosenthal:** Writing – review & editing, Supervision, Conceptualization. **Ron Moisseev:** Software. **Gil Gelbert:** Software. **Tamar Harary:** Writing – original draft, Visualization, Validation, Software, Methodology, Investigation, Formal analysis, Conceptualization.

## Consent for publication

Not applicable.

## Consent to participate

Not applicable.

## Helsinki approval

202–2024.

## Declaration of Competing Interest

The authors declare the following financial interests/personal relationships which may be considered as potential competing interests: Tamar Harary reports financial support was provided by Israel Innovation Authority. If there are other authors, they declare that they have no known competing financial interests or personal relationships that could have appeared to influence the work reported in this paper.

## Data Availability

Data will be made available on request.
