# Supplementary material for: In vivo optical-resolution optoacoustic flow cytometry in the oral mucosa using a transparent silicon-photonics acoustic detector (SPADE)
Source: Photoacoustics. 2026 Jul 30;51:100866. doi: 10.1016/j.pacs.2026.100866 (PMC13453587; doi:10.1016/j.pacs.2026.100866)
Supplement: Supplementary file 1 [file mmc1.docx]

Supplementary Information for:

***In vivo* optical-resolution optoacoustic flow cytometry in the oral mucosa using a transparent silicon-photonics acoustic detector (SPADE)**

Contents
Supplementary Note 1. Wafer transfer procedure

Supplementary Note 2. RBC preparation
Supplementary Note 3. Laser power at the capillary
Supplementary Note 4. Safety considerations

Supplementary Note 5. Laser wavelength stabilization for in-vivo measurements

*S1. Wafer transfer procedure*

The fabrication of the transparent SPADE followed the multistep process illustrated in Fig. S1. Both the silicon-photonics chip and the glass carrier were first cleaned in piranha solution (H₂SO₄:H₂O₂ 30 wt%, 2:1) to remove organic and biological residues. The sensor was then bonded to a 100-µm-thick glass substrate (JGS2 Quartz Discs**,** MICQ) using benzocyclobutene (BCB) adhesive (Cyclotene 3022-57), followed by vacuum baking through multiple temperature stages to fully cure the bonding layer (Fig. S1.2-3). After bonding and flipping the structure, the silicon layer was removed using deep reactive ion etching (Versaline, Plasma-Therm) with a CHF₃/O₂ gas mixture (Fig. S1.5). The SiO₂ cladding above the SiN waveguides was subsequently thinned in two stages. In the first thinning stage, the oxide was patterned using a positive photoresist mask (AZ1518, MicroChemicals GmbH) combined with a chrome mask fabricated with a laser writer (DWL 66+, Heidelberg instruments). The pattern was aligned using a mask-aligner system (MA6 KARL SUSS), leaving only the corner region of the chip exposed. The exposed corner region serves as a reference area for monitoring the etch progress and calibrating the oxide removal, enabling precise control of the residual SiO₂ thickness above the SiN waveguide. A short, buffered oxide etch (BOE 1:6, 3 min) selectively removed approximately 300 nm of SiO₂ from the exposed region, establishing a height reference relative to the rest of the layers (Fig. S1.6-8). In the second thinning stage (Fig. S1.9-10), a different lithographic mask was used to protect only the grating-coupler region while exposing the waveguide area to further oxide removal.

Controlled HF etching cycles were then performed consisting of short BOE etching cycles followed by rinsing and thickness monitoring using a profilometer (Bruker). The cycles continued until the non-critical reference region first became exposed. Based on the measured height difference between the reference and the protected waveguide regions, a final etch duration was calculated to thin the SiO₂ above the SiN resonator to the desired thickness of several tens of nanometers.

After completing the thinning process, a PDMS layer (Sylgard 184, Dow) was applied as a compliant acoustic cladding (Fig. S1.11). Polarization-maintaining fibers were coated on their exposed end facets with a 150 nm thick gold layer using an electron-beam evaporator (Evatec) to enhance thermal stability during operation. The gold-coated fibers were then aligned and bonded to the grating couplers (Fig. S1.12). The resulting transparent SPADE devices exhibited a fiber-to-fiber insertion loss of 19 dB at 1530 nm and a quality factor (Q-factor) of approximately 2.5 × 10⁵ for the central transmission resonance.

**Supplementary Fig. S1. Fabrication steps of the transparent SPADE.** (1) SiN-on-insulator chip prior to processing. (2-3) Surface cleaning and bonding to a 100-µm-thick glass substrate using a BCB layer, followed by vacuum backing. (4) Bonded assembly after curing and substrate flipping to expose the silicon handle wafer. (5) Removal of the silicon handle wafer by deep reactive ion etching (DRIE), followed by partial removal of the SiO₂ layer using reactive ion etching (RIE). (6-7) Photoresist coating and lithographic patterning using chrome masks to define a reference region for controlled over-etching, later used as a height calibration feature. (8) Initial buffered oxide etch (BOE, 1:6) forming an approximately 300-nm reference step. (9-10) Second lithography step to protect the waveguide couplers, followed by controlled hydrofluoric acid (HF) etching cycles to reduce the SiO₂ thickness to several tens of nanometers above the SiN waveguides. (11) Deposition of a PDMS (Sylgard 184) cladding layer. (12) Fiber alignment and bonding to the grating couplers.

*S2. RBC preparation and characterization*

Red blood cells (RBCs) were obtained as single-donor human RBC samples purchased from Almog Diagnostics (IWB3ALS40ML). Donor informed consent was obtained by the supplier, and all donor screening and health-related testing were performed in accordance with applicable clinical and ethical standards to ensure sample suitability. The blood samples were treated with ethylenediaminetetraacetic acid (EDTA) to prevent coagulation. All subsequent handling and processing of the samples were conducted in compliance with institutional ethical guidelines for the use of human biological materials.

Before performing an experiment, the blood was centrifuged for 5 minutes at 25 °C at a rotation speed of 3000 rpm to isolate the RBC fraction. The collected cells were then diluted at a 1:4 ratio (one-part RBCs to four parts saline). This dilution was necessary to reduce the risk of cell aggregation and to prevent clogging of microfluidic tubing during the experiments. The diluted RBC preparation was subsequently used for all microfluidic flow measurements described in this study.

To ensure stable flow and to eliminate air bubbles prior to measurements, the fluidic delivery system was carefully assembled and primed. RBC flow was delivered using micro medical tubing (LDPE, 95 durometers, 0.034″ I.D. × 0.052″ O.D.), connected via stainless steel dispensing needles with Luer-lock connections (20 gauge, 1.5″ needle length). A three-way T-connector (Dispoflex 3-Way Stopcock, white) was incorporated into the tubing assembly to allow controlled flushing and redirection of flow. At the beginning of each experiment, saline was first infused through the tubing up to the T-connector and diverted to an auxiliary outlet to remove trapped air from the main line. Saline was then flushed through the microfluidic phantom to verify continuous, bubble-free flow. Once stable flow conditions were confirmed, the diluted RBC sample was introduced following the same procedure, initially diverted through the T-connector and only subsequently directed into the phantom. This priming protocol effectively prevented air bubbles and ensured uninterrupted flow during all experiments.

*S2.1 Optical and optoacoustic event-rate comparison in the phantom*

To provide additional support for the interpretation of the phantom measurements, optical and optoacoustic event rates were compared under comparable phantom conditions in subsequent measurements. Because the available optical camera was not optimized for high-speed cell counting, the frame rate could be increased only to approximately 80 Hz, which was achieved by reducing both the acquisition window size and the exposure time together with adjustment of the illumination conditions to preserve RBC visibility. A typical optical image of an RBC is shown in Fig. S2a, where the RBC appeared smeared due to the low frame rate. Fig. S2b shows the same image after background subtraction, highlighting the RBC. As the figure shows, while this configuration enabled visualization of RBC motion, it also increased image noise and reduced the optical signal-to-noise ratio. To further enable optical observation of individual RBC passages at a low frame rate, the syringe pump was switched off and measurements were performed over time under diffusive-flow conditions, resulting in sufficiently slow RBC motion for manual optical counting. Under these conditions, the optical measurements yielded average event rates of approximately 23, 10, and 8 events/s (Fig. S2c). The measurement was repeated with the optoacoustic setup, revealing rates of approximately 35, 15, and 12 events/s (Fig. S2d). The difference between the measurement results may be explained by the natural variation in the phantom in the case of diffusive flow between two independent measurements and limitations of the optical setup. Nonetheless, the qualitative behavior of reduction in the flow rate over time, obtained in both modalities, provides support for the interpretation of the detected optoacoustic transients as RBC-related events.

The residual differences between the optical and optoacoustic event rates are likely due to several factors. First, the optical measurements were performed at a frame rate of approximately 80 Hz, whereas the optoacoustic measurements were acquired at 1 kHz, resulting in substantially different temporal resolutions. Second, maximizing the optical frame rate required reduction of the acquisition window size and exposure time, together with specific illumination conditions to preserve RBC visibility, which increased noise and reduced the optical signal-to-noise ratio. Third, to slow the flow sufficiently for optical observation, the pump was switched off and the measurements were performed over time under diffusive-flow conditions. Finally, the optoacoustic event rate was normalized based on the estimated effective RBC interaction length relative to the optical spot size, and this normalization is inherently approximate. Accordingly, the observed agreement should be regarded as partial validation rather than as a definitive quantitative ground-truth comparison.

**Supplementary Fig. S2. Comparison of optical and optoacoustic event rates in the phantom.**
(a) Representative raw optical frame, showing that an individual RBC can still be identified despite the limited image quality obtained under the current high-frame-rate optical acquisition conditions. (b) Corresponding background-subtracted image. (c) Mean optical event rates measured under three comparable phantom conditions, with error bars indicating standard deviation. The optical measurements yielded average event rates of approximately 23, 10, and 8 events/s. (d) Mean optoacoustic event rates measured under the same comparable phantom conditions, with error bars indicating standard deviation. The corresponding optoacoustic measurements yielded approximately 35, 15, and 12 events/s. Although the measurements were not acquired simultaneously, the comparable magnitudes and consistent ordering across conditions provide partial support for the interpretation of the detected optoacoustic transients as RBC-related events.

*S2.2 Acoustic angular response of the transparent SPADE*

To further support the discussion of detector positioning tolerance, the acoustic angular response of the transparent SPADE used in the present study was characterized using the optoacoustic data used to produce Fig. 4e. Specifically, the data included the response of the SPADE element to an optoacoustic point source scanned over a plane with a distance of 0.75 mm. The peak-to-peak amplitude of the distance-scaled signal was evaluated as a function of angle, and the result is shown in Supplementary Fig. S3, for the two scan directions. The results show half-maximum acceptance angle of approximately ±50° and ±7°, respectively.

**Supplementary Fig. S3. Measured angular response of the transparent SPADE used in the present study.** The peak-to-peak amplitude of the distance-scaled signal is shown as a function of angle for the two principal axes of the detector over 140 MHz bandwidth. The dashed line indicates the half-maximum level. The transverse-axis response (left) demonstrates a broad angular response of approximately ±50°, whereas the longitudinal-axis response (right) exhibits a narrower acceptance angle of approximately ±7°, consistent with the elongated geometry of the SPADE element.

*S3. Laser power at the capillary*

The average optical power measured at the output of the single-mode fiber and collimator assembly using a calibrated power meter (918D-SL-OD2R sensor and 1919-R power meter, Newport) was approximately 0.8 mW. After transmission through the dichroic beam splitter, which introduced an estimated loss of about 3%, the power was reduced to approximately 0.776 mW. Additional attenuation occurred upon transmission through the transparent SPADE due to scattering and beam distortion. Based on experimental characterization, the sensor transmission was approximately 60%, resulting in an estimated optical power of ~0.47 mW incident on the tissue surface.

Further attenuation occurs due to optical scattering in the oral mucosa before the excitation light reaches the superficial capillaries. The oral mucosa is commonly modeled as a superficial avascular epithelial layer overlying a stromal layer that contains capillary network ^S1^. At a wavelength of 532 nm, the reported optical scattering coefficient of the epithelial layer is approximately 100 cm⁻¹, while the stromal layer exhibits higher scattering ^S1^. Reflectance-based measurements in oral and sinus mucosa have reported an effective bulk scattering coefficient of approximately 170 cm⁻¹, assuming an anisotropy factor of g = 0.9 ^S2^.

The thickness of the epithelial layer in the lip mucosa has been reported to have a mean value of approximately 199 µm with a standard deviation of 113 µm ^S3^. For a scattering coefficient of 100 cm⁻¹, this thickness range corresponds to ballistic photon transmission values between approximately 0.42 (thin epithelium) and 0.04 (thick epithelium). Since the optical focus relies on the un-scattered ballistic photons, these values describe the attenuation experienced by the focused beam. Accordingly, the optical power reaching the capillary is expected to vary between 0.019 mW to 0.2 mW.

*S4. Safety considerations*

Based on the estimated optical power reaching the capillary, the corresponding estimation for the pulse energy ranged from 19 nJ (thick epithelium) to 200 nJ (thin epithelium). The beam was focused to a spot diameter lower than 4 µm, resulting in an illuminated area of about 1.26 × 10⁻⁷ cm² and an estimated optical fluence in the range 0.15-1.59 J·cm⁻² at the capillary. This fluence is well below reported photodamage thresholds for red blood cells at a wavelength of 532 nm, which have been shown to be approximately 5 J·cm⁻² ^S4^. Photoacoustic-based studies have further demonstrated safe RBC interrogation at fluence levels below this threshold without observable cellular damage ^S5^. Throughout the *in vivo* experiments, no visible tissue damage, signal degradation, or changes in red blood cell behavior were observed. These results confirm that the optical excitation conditions used in this study provide a sufficient safety margin while enabling reliable single-cell optoacoustic detection in superficial inner lip capillaries.

*S5. Laser wavelength stabilization for in-vivo measurements*

Due to the high-quality factor of the resonator and the large photo-elastic and thermo-optic coefficients of polydimethylsiloxane (PDMS), small pressure or temperature perturbations at the optoacoustic sensor can induce shifts in the resonator’s resonance wavelength, leading to a mismatch with the interrogation laser and consequent signal loss. During the *in vivo* measurements, such perturbations mainly originated from mechanical pressure applied during contact with the lip tissue and from temperature variations associated with sensor–tissue contact and the application of ultrasound coupling gel.

To ensure stable sensor operation, a feedback-based wavelength-locking scheme was implemented to maintain alignment between the laser and the resonator. The resonance wavelength exhibited both short-range rapid fluctuations (typically < 100 pm) and slower wide-range drifts (typically < 1 nm), necessitating a two-tier stabilization approach. The interrogation laser (TSL-570, Santec) provides coarse digital tuning with a resolution of 0.1 pm, as well as fine continuous analog tuning with a span of 120 pm and a maximum slew rate of 30 nm/s. Rapid resonance fluctuations were compensated using a Pound–Drever–Hall locking scheme implemented via a VHDL-based controller (Moku:Go, Liquid Instruments), while slower drifts were tracked using a MATLAB-based digital tuning loop, ensuring that the analog feedback remained within its tuning range.

Supplementary References

[S1] S.L. Jacques, Optical properties of biological tissues: a review, *J. Biomed. Opt.* **17** (2012) 107003. <https://doi.org/10.1117/1.JBO.17.10.107003>

[S2] A.N. Bashkatov, E.A. Genina, V.I. Kochubey, V.V. Tuchin, Optical properties of human skin, subcutaneous and mucous tissues in the wavelength range from 400 to 2000 nm, *J. Phys. D: Appl. Phys.* **38** (2005) 2543–2555. <https://doi.org/10.1088/0022-3727/38/15/004>

[S3] T. Igarashi, Y. Oda, K. Harada, et al., Thickness of the oral mucosa of the lip measured by optical coherence tomography, *J. Periodontal Res.* **56** (2021) 115–123.

[S4] D.O. Lapotko, V.P. Zharov, Spectral evaluation of laser-induced cell damage with photothermal microscopy, *Lasers Surg. Med.* **36** (2005) 22–30. <https://doi.org/10.1002/lsm.20110>

[S5] W.-J. Li, Y. Liu, X. Zhang, et al., Real-time red blood cell counting and osmolarity analysis using a photoacoustic-based microfluidic system, *Photoacoustics* **21** (2021) 100230. https://doi.org/10.1016/j.pacs.2020.100230
